# Transmission blocking potency and immunogenicity of a plant-produced Pvs25-based subunit vaccine against *Plasmodium vivax*

**DOI:** 10.1016/j.vaccine.2016.05.007

**Published:** 2016-06-14

**Authors:** A.M. Blagborough, K. Musiychuk, H. Bi, R.M. Jones, J.A. Chichester, S. Streatfield, K.A. Sala, S.E. Zakutansky, L.M. Upton, R.E. Sinden, I. Brian, S. Biswas, J. Sattabonkot, V. Yusibov

**Affiliations:** a Department of Life Sciences, Sir Alexander Fleming Building, Imperial College London, Imperial College Road, South Kensington, London SW7 2AZ, UK; b Fraunhofer USA Center for Molecular Biotechnology, Newark, DE, USA; c Jenner Institute, The University of Oxford, Roosevelt Road, Oxford OX9 2PP, UK; d Department of Entomology, Armed Forces Research Institute of Medical Sciences, Bangkok 10400, Thailand

**Keywords:** Transmission blocking vaccine, Malaria, Plant-produced antigen, *Plasmodium*, Pvs25, Subunit vaccine

## Abstract

Malaria transmission blocking (TB) vaccines (TBVs) directed against proteins expressed on the sexual stages of *Plasmodium* parasites are a potentially effective means to reduce transmission. Antibodies induced by TBVs block parasite development in the mosquito, and thus inhibit transmission to further human hosts. The ookinete surface protein P25 is a primary target for TBV development. Recently, transient expression in plants using hybrid viral vectors has demonstrated potential as a strategy for cost-effective and scalable production of recombinant vaccines. Using a plant virus-based expression system, we produced recombinant P25 protein of *Plasmodium vivax* (Pvs25) in *Nicotiana benthamiana* fused to a modified lichenase carrier protein. This candidate vaccine, Pvs25-FhCMB, was purified, characterized and evaluated for immunogenicity and efficacy using multiple adjuvants in a transgenic rodent model. An *in vivo* TB effect of up to a 65% reduction in intensity and 54% reduction in prevalence was observed using Abisco-100 adjuvant. The ability of this immunogen to induce a TB response was additionally combined with heterologous prime-boost vaccination with viral vectors expressing Pvs25. Significant blockade was observed when combining both platforms, achieving a 74% and 68% reduction in intensity and prevalence, respectively. This observation was confirmed by direct membrane feeding on field *P. vivax* samples, resulting in reductions in intensity/prevalence of 85.3% and 25.5%. These data demonstrate the potential of this vaccine candidate and support the feasibility of expressing *Plasmodium* antigens in a plant-based system for the production of TBVs, while demonstrating the potential advantages of combining multiple vaccine delivery systems to maximize efficacy.

## 1 Introduction

Malaria is a global disease caused by parasites of the genus *Plasmodium*. An estimated 207 million cases of malaria are reported annually, causing approximately 627,000 deaths [1], [2]. Of the five species of malaria parasites that infect humans, *Plasmodium falciparum* and *Plasmodium vivax* lead to the greatest burden of disease. While *P. falciparum* is responsible for the majority of malaria-linked deaths, *P. vivax* can cause relapses months after the first infection [4] caused by hypnozoites, and require specialized treatment, e.g. primaquine [2], [3], [4]. *P. vivax* is the most widely distributed human malaria parasite, with 2.5 billion people at risk of infection, and 80−300 million cases per annum [1], [3]. Multiple factors, including the appearance of chloroquine-resistant *P. vivax* [5], lack of practical alternatives to primaquine, combined with increasing global temperatures [6], [7], have raised concerns related to increased risks of *P. vivax*-induced disease.

No licensed vaccine for malarial prophylaxis is currently available [8], [9]. Chemotherapy is the only clinically available treatment for confirmed infection; however, recurring drug resistance reduces the efficiency of anti-malarials [4]. The importance of vaccines against *P. vivax* is well understood, but a lack of long-term *in vitro* culture systems and suitable animal models have hindered developmental advances.

Malaria transmission-blocking (TB) vaccines (TBVs) show promise as a method to reduce transmission. Briefly, antibodies produced within an individual in response to vaccination are ingested by the mosquito along with gametocytes, during a bloodmeal. These antibodies prevent parasite development in the mosquito midgut by binding to surface proteins of the sexual stages, impeding further transmission [10], [11]. One of the primary targets for TBV development is the P25 protein, expressed predominantly on the surface of the *Plasmodium* zygote and ookinete [12]. P25 is characterized by the presence of epidermal growth factor (EGF)-like motifs, multiple cysteine residues and a complex tertiary structure [13], making it challenging to produce recombinant protein with appropriate conformation. Previous studies that have successfully expressed *P. vivax* P25 (Pvs25) in native conformation have been limited to small-scale studies, with “classical” recombinant expression systems [15], [16], [17] or using multiple viral delivery systems [18], [19], [39].

Previously, studies have examined the potential of utilizing plants as a cost-effective and scalable platform for vaccine production [20], [21]. *Nicotiana benthamiana* has been used in conjunction with a hybrid plant virus vector-based expression system [22] to produce subunit vaccine candidates against influenza, plague and anthrax [23], [24], [25], [26], [27], [28]. In related studies, this system has produced soluble versions of *P. falciparum* P25 (Pfs25), either as stand-alone proteins or as fusions to the modified lichenase carrier molecule (LicKM). In mice and rabbits, fusion and non-fusion versions of plant-produced Pfs25 elicited high titers of anti-Pfs25 antibodies when administered with Alhydrogel as an adjuvant. These antibodies demonstrated potent TB activity [29], [30].

Here, a Pvs25-LicKM fusion protein (Pvs25-FhCMB) was produced using *N. benthamiana* as an expression host. We examined efficacy of this candidate vaccine by performing a head-to-head comparison of induced TB potency following mouse immunization with recombinant Pvs25-FhCMB in the presence of two clinically relevant adjuvants: Alhydrogel, a common aluminium hydroxide wet gel suspension, and Abisco-100, a non-toxic saponin-based adjuvant, and compared these to immunization with a lead adenoviral vaccine platform. Recently, the development of viral vectored blood-stage malaria vaccines has shown that high-level antibody responses can be induced by the adenovirus in mice [31], [32], rabbits [33], [34] and rhesus macaques [35]. Studies on vaccine candidates AMA1 and MSP have demonstrated that this regimen is safe and immunogenic [36], [37]. Previous experiments involving immunization of mice with adenovirus expressing Pfs25 or Pvs25 have led to antibodies exhibiting TB efficacy [38], [39], [49].

We show that immunization of mice with Pvs25-FhCMB elicited effective Pvs25-specific humoral immune responses and significant TB activity. The ability of antiserum generated from each immunization regime to recognize native Pvs25 was examined by immunofluorescence assay (IFA). TB activity was assessed *in vivo*, using a transgenic rodent malaria parasite *P. berghei* (Pvs25DR3) expressing Pvs25, enabling rapid, safe and cost-effective examination of anti-Pvs25 responses [18], [19], [40]. We additionally examined the benefit of a heterologous prime/boost regimen by priming animals with Pvs25-FhCMB followed by boosting with recombinant chimpanzee adenovirus expressing Pvs25 (ChAd63-Pvs25). Efficacy was examined by direct feeding assay (DFA), and in direct membrane feeding assay (DMFA) against *P. vivax* field samples. Maximal efficacy was observed when combining adenoviral and plant-derived immunogens in a single regime. Our data indicate that *Nicotiana*-produced Pvs25 antigen can induce significant TB responses *in vivo* and *ex vivo*, and that this technology could be used as part of a successful TBV immunization regime, either alone, or in combination with other delivery systems.

## 2 Materials and methods

### 2.1 Pvs25-FhCMB cloning and expression

**Fig. 1:**
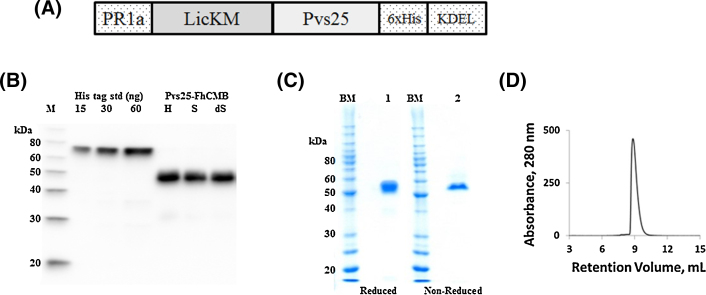
Design, expression and purification of Pvs25-FhCMB. (a) Schematic representation of the Pvs25-FhCMB expression construct showing positions of the PR-1a leader sequence, LicKM carrier protein, Pvs25 antigen and C-terminal 6xHis tag and KDEL. (b) Western blot showing expression of Pvs25-FhCMB in unclarified homogenate (H), soluble (S) and detergent-solubilized (dS) fractions. Molecular weight markers (M) are MagicMark standards (Invitrogen). (c) SDS-PAGE (10%) analysis of Pvs25-FhCMB (2 μg load) stained with Coomassie. Pvs25-FhCMB was run under denatured, reducing conditions (1) or denatured, non-reducing conditions (2). Molecular weight markers (M) are BenchMark standards (Invitrogen). (d) Analytical SEC (Zenix 300) of purified Pvs25-FhCMB (200 μg load).

Pvs25 protein sequence corresponding to Ala23-Leu195 (NCBI accession XP_001608460) was codon optimized for plants expression (GeneArt®, Germany). The sequence was fused to the C-terminus of LicKM (NCBI accession number ABG78599) [22], containing pathogen-related-protein 1a signal peptide (PR-1a) [(NCBI accession BAA14220)] at the N-terminus, the hexa-histidine (6xHis) tag and the endoplasmic reticulum retention signal at the C-terminus, to yield Pvs25–FhCMB (Fig. 1a). The gene was cloned into the TMV-based hybrid expression vector pGRD4 [22], [27]. Vector was introduced into *Agrobacterium tumefaciens* GV3101 by electroporation, and vacuum infiltrated into leaves of six-week-old hydroponically grown *N. benthamiana* as described previously [22], [27].

### 2.2 Pvs25-FhCMB protein purification and characterization

*N. benthamiana* plants expressing Pvs25-FhCMB were harvested at 6 dpi and stored at ≤-60 °C. Plants were homogenized, extracted with Triton X-100 (0.5%), centrifuged (16,000 × g, 15 min, 4 °C) and filtrated (Sartopore 2, 0.45/0.2 μm) prior to loading onto IMAC resin (Ni-Sepharose Fast-Flow, GE Healthcare). Proteins was eluted and loaded onto a CaptoQ column (GE Healthcare). Eluted fraction was concentrated 10-fold by centrifugation (Centricon-70, 30 kDa MWCO) and aliquots were frozen at ≤-60 °C. LicKM was expressed as described previously [29], [30].

SDS-PAGE was performed on a 10% acrylamide gel and Coomassie stained. For Western blot, samples were blocked with I-Block (Applied Biosystems). Blots were developed using anti-4xHis mAb (Qiagen). Protein concentration of Pvs25-FhCMB was determined by UV−vis spectrometry using the denaturing method of Edelhoch (1967) with the theoretical extinction coefficient of 73,960 M^−1^ cm^−1^. Analytical size exclusion chromatography was performed on a Zenix300 HPLC column (Sepax Technologies, Inc.).

### 2.3 Production of viral vector-based Pvs25 vaccines

Antigen sequence (excluding the glycosylphosphatidylinositol (GPI) anchor addition site), i.e. Ala23-Leu195 of Pvs25 (Sal I strain), was obtained from the NCBI protein database, codon optimized for expression in *Homo sapiens* (GeneArt®, Life Technologies, Germany), and the predicted native signal peptide replaced with the human tissue plasminogen activator signal peptide. Recombinant ChAd63, MVA vaccines and controls were generated [31], [38], [42] .

### 2.4 Production of recombinant Pvs25 (r-Pvs25) protein

Pvs25 antigen sequence was codon optimized for expression in *Homo sapiens*, subcloned into modified pENTR4-LPTOS TM [48] plasmid between a tPA leader and an affinity sequence encoding a biotin-acceptor peptide and a hexahistidine tag. Plasmids were transfected into 2 × 10^6^ cells/mL HEK293E cells. Soluble recombinant protein (r-Pvs25) was affinity-purified using a HiTrap HP nickel column (GE Healthcare, USA), followed by size exclusion chromatography using a Superdex 200 pg gel filtration column (GE Healthcare, UK) and confirmed by SDS-PAGE.

### 2.5 Immunization regimes

**Fig. 2:**
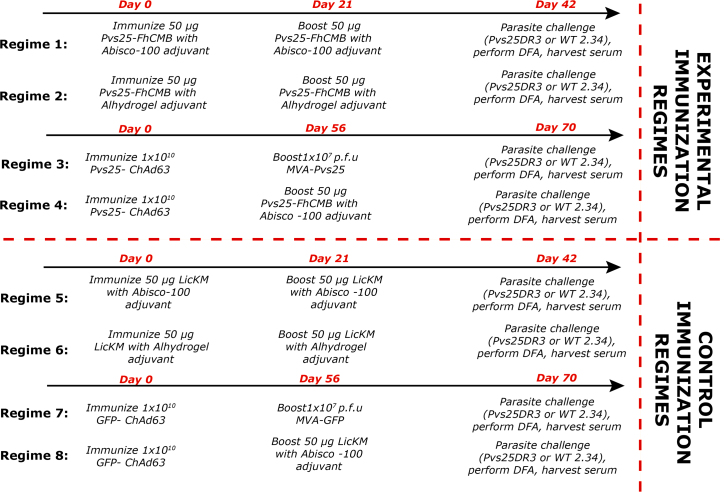
Anti-Pvs25 immunization regimes. Groups of 10 mice received each vaccine regime. In each individual regime, for DFA five mice were challenged with *P. berghei* Pvs25DR3 to assess for Pvs25-specific TB effects, and five mice were challenged with *P. berghei* 2.34 to control for non-specific TB effects. In regimes 1−4, mice were immunized to attempt to induce a Pvs25 response. In regimes 5−8, mice were immunized with carrier protein or empty vector controls. All immunizations were performed *i.m*.

Eighty mice (Female BALB/c, 6–8 weeks of age (Harlan, UK)) were divided into groups of 10 mice, each group used for an individual immunization regime (Fig. 2). Regimes 1−4 were experimental whereas regimes 5−8 were immunized (*i.m.*) with control immunogens.

In each group, five mice were challenged with Pvs25DR3 [40], and five were challenged with wild-type (WT) *P. berghei* (2.34). Transmission blockade observed with 2.34 was considered non-specific and not attributed to Pvs25 immunization.

For vaccination with Alhydrogel, 10 doses (50 μL per mouse) were prepared by combining 85 μL of Alhydrogel™ containing 850 μg of aluminium in Eppendorf tubes under aseptic conditions with 165 μL of TRIS buffer (20 mM Tris, pH 7.4, 0.9% NaCl). After 15 min, 250 μL of antigen solution was added to corresponding tubes, which were vortexed and incubated for 1 h. For Abisco-100™ adjuvant, recombinant protein was mixed with adjuvant, vortexed for 10 s and injected immediately (5 μL Abisco-100 per mouse in total vaccination volume). Viral-vectored vaccines were prepared in sterile, endotoxin-free PBS prior to immunization, with doses of 1 × 10^10^ viral particles of ChAd63 vaccines at week 0 and 1 × 10^7^ plaque forming units of MVA vaccines at week 8. Control mice were immunized with ChAd63 and MVA expressing GFP as an irrelevant antigen in place of Pvs25 (Ad-MVA GFP) [42]. For regimens where Pvs25-FhCMB protein was used for immunization and boost, sera were collected at days 0, 21 and 42. When initial immunization was performed with ChAd63, sera were collected at days 0, 56 and 70.

### 2.6 ELISA

Sera from mice were collected by tail-bleed prior to boost and challenge. Nunc-Immuno Maxisorp 96-well plates (NUNC) were coated with 100 ng recombinant protein (Pvs25FhCMB or r-Pvs25) overnight. Test sera was incubated for 2 h followed by goat anti-mouse IgG-AP secondary (Sigma), 1:3000. Plates were developed using pNPP and read at OD_405_ until end-point detection set for each antigen. For r-Pvs25 ELISA, control regime titres were subtracted from mean end-point titres. The cut-off for determining end-point titer was the mean OD value for pre-immune sera + three times the standard deviation.

### 2.7 Parasite maintenance and challenge/DFA

Routine maintenance of *P. berghei* was carried out as described previously [40], [41].

Prior to challenge, mice were PHz treated, and 3 days later infected i.p. with 10^6^ *P. berghei* ANKA 2.34 or *P. berghei* Pvs25DR3 [19], [40]. Three days post-infection, DFA was performed as described in Blagborough et al. [19], with >50 *Anopheles stephensi* mosquitoes allowed to feed on each mouse. Twelve days later, midguts were dissected, and oocyst prevalence and intensity recorded and inhibition, these values were compared to intensity and prevalence in mice immunized with appropriate controls.

### 2.8 DMFA

Peripheral blood was collected from *P. vivax* infected Thai volunteer patients. Ninety microliter of pooled serum from mice immunized with ChAd63u-Pvs25/Pvs25-FhCMB boost (regime 4) or mice immunized with ChAd63-GFP/LicKM boost (regime 8) were mixed with 90 μL of heat-inactivated AB serum from naive donors. Serum was mixed with *P. vivax-*infected blood cells (1:1 v/v ratio). The mixture was placed in a membrane feeder, 37 °C and *Anopheles dirus* A mosquitoes (Bangkok colony, Armed Forces Research Institute of Medical Sciences) were allowed to feed for 30 min. Mosquito maintenance and dissection were performed as described in Blagborough et al. [19]. Reductions in intensity and prevalence were assessed with respect to the relevant controls.

### 2.9 IFA

The ability of harvested sera to recognize native Pvs25 was assessed by IFA on Pvs25DR3 ookinetes as described in Sinden et al. [41].

### 2.10 Ethical statement

All procedures were performed in accordance with the UK Animals (Scientific Procedures) Act (PPL 70/7185) and approved by the Imperial College AWERB. The Office of Laboratory Animal Welfare Assurance for Imperial College covers all Public Health Service supported activities involving live vertebrates in the US (no. A5634-01). Protocol for blood collection from Thai patients (protocol # TMEC-11-033) was approved by Ethical Committees from the Faculty of Tropical Medicine, Mahidol University and Ministry of Public Health, Thailand.

### 2.11 Statistical analysis

Statistical analysis was performed using Graphpad Prism. For DFA and DMFA, significance was assessed using Mann–Whitney U (to examine differences in intensity) and Fisher's exact probability (to examine differences in prevalence). Parametric ELISA tests were assessed using *t*-test. *P*-values < 0.05 were considered statistically significant.

## 3 Results

### 3.1 Expression, purification and characterization of Pvs25-FhCMB

Pvs25-FhCMB expression was evaluated by Western blot of clarified extract (Fig. 1b). Solubility was examined using an anti-4xHis mAb by Western blot of total unclarified protein homogenate (H), total soluble protein (S) and total soluble protein extracted with 0.5% Triton X-100 (dS) (Fig. 1b). Densitometry analysis of Western blots indicated that Pvs25-FhCMB was ∼100% soluble.

Pvs25-FhCMB was recovered to >90% purity of TP using a two-column chromatography approach consisting of IMAC followed by anion exchange chromatography (Capto Q). SDS-PAGE indicates a single prominent band at approximately 55 kDa (Fig. 1c). When Pfs25-FhCMB was run in non-reducing buffer, a single band, migrating at a similar molecular weight is observed. Analysis by analytical SEC indicates a single prominent peak eluting at a size equivalent to a 45 kDa standard (Fig. 1d), in good agreement with the theoretical molecular weight of 46.4 kDa. Both the non-reduced SDS-PAGE and the single SEC peak indicate Pfs25-FhCMB is a non-aggregated, monomeric protein.

### 3.2 Immunogenicity of Pvs25-FhCMB and induction of target-specific antibodies

To examine immunogenicity, pre-boost and end-point sera were analyzed by ELISA against recombinant Pvs25-FhCMB (comprising LicKM) following immunization as outlined in Fig. 2. Sera were taken from individual mice (five per group) and analyzed for Pvs25-FhCMB-specific responses. No antibody responses against Pvs25-FhCMB were detected in day 0 sera.

**Fig. 3:**
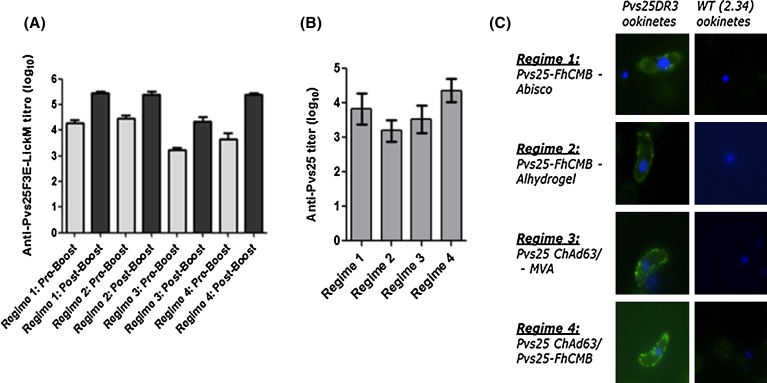
Induction of antibody following immunization with Pvs25-FhCMB. The ability of different immunization regimes to generate Pvs25-specific antibody responses was tested by ELISA against recombinant Pvs25-FhCMB and IFA against Pvs25DR3 ookinetes. (a) Pre-boost and end-point titers of anti-Pvs25-FhCMB in serum. Bars show mean titers from five mice. Light grey = pre-boost, dark grey = post-boost. Pre-immune serum did not recognize recombinant Pvs25-FhCMB. Error bars represent SEM. (b) End-point titers of anti-r-Pvs25 serum. Bars show mean titers from five mice. Error bars represent SEM. (c) IFA against Pvs25DR3 ookinetes. Ability of generated serum to recognize native Pvs25 on the surface of transgenic Pvs25DR3 ookinetes was assessed by immunofluorescence on fixed, non-permeabilized parasites probed with anti-serum from each regime. To control for non Pvs25-specific signal, IFA was performed against WT 2.34 ookinetes. Each panel shows an overlay of anti-Pvs25 signal (green) and DNA labeled with DAPI (blue). IFA with non-Pvs25 derived (control) serum from regimes 5−8 resulted in no significant staining.

Analysis of responses following Pvs25-based immunization detected anti-Pvs25 antibody both pre- and post-boost for all regimes tested, with higher end-point titers detected post-boost (Fig. 3a). There was a significant increase in titers following booster immunizations in regimes 1, 2 and 4 (*p* < 0.05), with the most pronounced increase detected in regime 4, and a non-significant increase in titers following boost in regime 3 (*p* = 0.145). The highest mean titer was observed in regime 1 (Pvs25-FhCMB + Abisco-100). The lowest anti-Pvs25-FhCMB end-point titers were exhibited with regime 3 (Pvs25-ChAd63 + MVA). Differences detected between mean end-point titers from regimes 1, 2 and 4 were not statistically significant (*p* > 0.05). End-point titers resulting from immunization with Pvs25-ChAd63/MVA (regime 3) were significantly lower than those detected in all other groups (compared to regime 1: *p* < 0.0001; compared to 2 (*p* = 0.0163); compared to 4 (*p* = 0.0003). To assess end-point response to Pvs25 *per se*, as opposed to Pvs25-LicKM conjugate, ELISA was carried out against recombinant r-Pvs25 (Fig. 3b). The lowest mean end-point titers were observed against serum from regime 2, whereas the highest was observed from regime 4; however, differences detected between end-point titers from all regimes were not statistically significant (*p* > 0.05).

To confirm the induction of Pvs25-specific antibodies that recognize native protein, we performed IFA on *P. berghei* Pvs25DR3 ookinetes using pooled end-point serum. Specific-specific staining was observed using sera from immunization regimes 1, 2, 3 and 4 (Fig. 3c), confirming the ability of generated serum to recognize native Pvs25. Staining was not observed with sera from any regime against WT ookinetes, or with sera derived from control regimes (i.e. regimes 5, 6, 7 and 8).

### 3.3 Evaluation of *in vivo* TB activity (DFA)

For examination of transmission blockade *in vivo*, five immunized mice per challenge group were infected with Pvs25DR3 [40], and five mice infected with WT *P. berghei*. Three days later, DFA was performed (Table 1a). Transmission blockade was assessed as reduction in intensity and prevalence with respect to the relevant non-Pvs25 control regimes (i.e. regime 1 is controlled by regime 5, regime 2 by regime 6, regime 3 by regime 7 and regime 4 by regime 8–Table 1b). For each Pvs25-derived regime, a TB effect was observed following infection with *P. berghei* Pvs25DR3. Significant reduction in intensity was observed with regimes 1, 2 and 4, and significant reduction in prevalence was observed with all Pvs25-regimes. The highest efficacy in terms of both intensity and prevalence was observed following immunization with Pvs25-ChAd63, boosted with Pvs25F3E-LicKM/Abisco-100 (regime 4), where a mean reduction in intensity/prevalence of 74.5%/68.3%, respectively was achieved. Direct correlation between anti-Pvs25 titer and transmission-blocking efficacy in individual mice was not observed with any significance. As previously demonstrated [18], [19], [40], challenge with WT *P. berghei* gives rise to higher oocyst numbers than observed with Pvs25DR3. There was no detectable TB effect following WT *P. berghei* infection, indicating specific anti-Pvs25-derived transmission blockade.

### 3.4 Evaluation of *ex vivo* TB activity (DMFA)

**Fig. 4:**
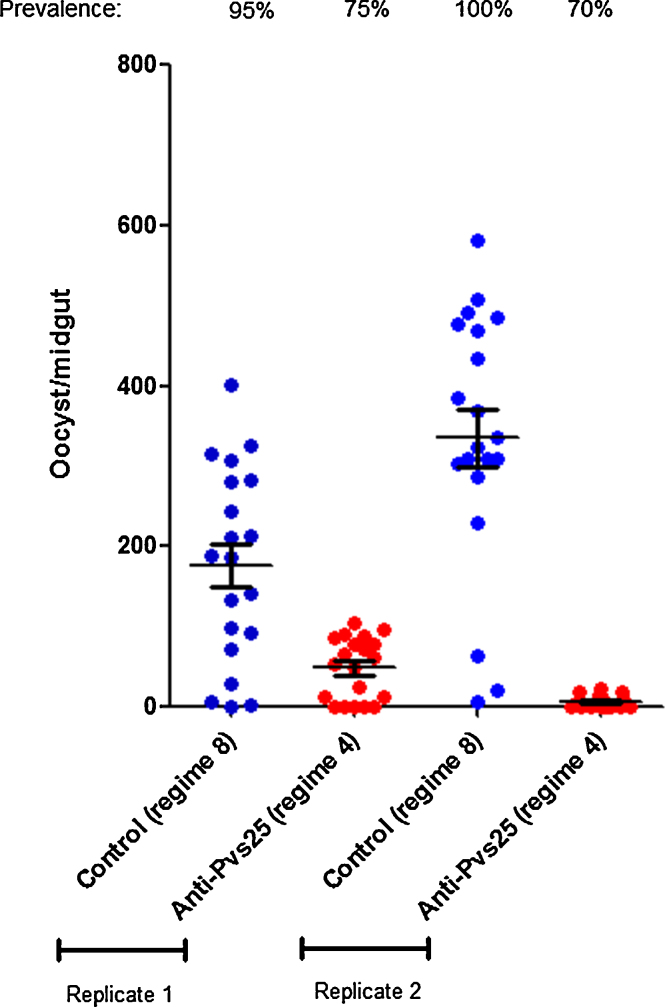
*Ex vivo* transmission-blocking efficacy of serum generated by Pvs25-ChAd63 prime and Pvs25-FhCMB/Abisco-100 boost using in the DMFA. Pooled serum sera from mice immunized with ChAd63u-Pvs25 and boosted with Pvs25-FhCMB (regime 4) or mice immunized with ChAd63-GFP and boosted with LicKM (regime 8, negative control) − were mixed 1:1 with heat-inactivated normal human AB serum prepared from malaria-naive Thai donors. Diluted serum was then mixed with *P. vivax*-infected blood cells (1:1 v/v ratio). Duplicate feeds were performed. Individual data points represent the number of oocysts found in individual mosquitoes 12 days post feed. Horizontal bars indicate mean intensity of infection, whilst error bars indicate S.E.M. within individual samples.

Serum collected from mice primed with Pvs25-ChAd63 and boosted with Pvs25-FhCMB/Abisco-100 (regime 4), was pooled and evaluated in DMFA. Results showed that the intensity and prevalence of oocyst infection on the mosquito midgut was profoundly reduced in comparison with serum collected from the control group immunized with GFP-ChAd63 and boosted with LicKM/Abisco-100 (regime 8) (Fig. 4). Over two replicate experiments, an overall 85.3% reduction in infection intensity and a 25.6% reduction in prevalence were observed. Reduction in both intensity and prevalence were significant compared to control feeds (*p* = 0.0001 and *p* = 0.0033, respectively).

## 4 Discussion

Future control of malaria will require a multidimensional approach. Both the WHO and the Malaria Eradication Research Agenda have previously set as a core goal for any malaria vaccine program the need to reduce transmission and morbidity [43]. TBVs are one potential component of this approach. Anti-malarial TB immunity can be mediated by antibodies that inhibit parasite development in the mosquito midgut [43], [44], [45], [46], [47].

We have previously reported on the production of Pfs25 variants in *N. benthamiana* [29]. In that study, the presence of *N*-linked glycans impaired the induction of TB activity by the recombinant non-fusion version of Pfs25. To increase immunogenicity, Pfs25 lacking *N*-glycosylation sites was fused to modified lichenase carrier molecule, LicKM [30]. The resulting protein induced high titers with long-lasting TB activity in mice. These results indicated the utility of plant-derived Pfs25 as a TBV, and the importance of appropriate carrier molecules to enhance immunogenicity.

Here, in a parallel study, we describe the production and purification of soluble, Pvs25 recombinant antigen (Pvs25-FhCMB), using a viral plant-based system. Similar to Pfs25, the Pvs25 sequence contains 22 cysteines, but unlike the Pfs25 sequence Pvs25 does not contain any putative *N*-linked glycosylation sites. Induction of functional TB antibody responses against Pvs25 was assessed, following immunization with four different regimes: 1). Pvs25-FhCMB with Abisco-100 adjuvant; 2). Pvs25-FhCMB with Alhydrogel; 3). Viral delivery of Pvs25 (Pvs25-ChAd63) with an MVA boost (i.e. classical Adeno-MVA ‘prime-boost’); and 4). A combination of adenoviral delivery (Pvs25-ChAd63) with a Pvs25-FhCMB recombinant protein boost. Ability of each regime to produce antibody capable of recognizing native protein and exhibit TB efficacy (*in vivo* and *ex vivo*, with field samples) was examined.

Immunization resulted in immunogen-specific responses against recombinant Pvs25-FhCMB and r-Pvs25 in all immunization regimes tested. As expected, a booster dose significantly increased the antibody titer against Pvs25-FhCMB in each regime. The highest mean titer was observed with regime 1 (prime/boost with Pvs25-FhCMB/Abisco-100), whereas the lowest anti-Pvs25-FhCMB end-point titers were elicited by regime 3 (Pvs25-ChAd63/MVA). Recognizing that ELISAs against the Pvs25-FhCMB recombinant protein included significant responses to LicKM (Fig. 3a outlines responses to Pvs25-FhCMB, comprising LickM), we examined responses against Pvs25 specifically (Fig. 3b). Under these conditions, the highest titer was observed with regime 4 (Pvs25-ChAd63/Pvs25-FhCMB/Abisco-100), and the lowest with regime 2 (Pvs25-FhCMB/Alhydrogel). However, when objectively comparing differences in responses, it should be noted that no significant difference could be detected between regimes. It should be stated that no detectable correlation between anti-Pvs25 titer and transmission-blocking efficacy in individual mice was observed, supporting the hypothesis that antibody titer is not necessarily the key parameter for predicting TBV efficacy. It is likely that other additional qualitative changes, such as isotype switching and affinity maturation, will significantly impact the biological activity and effector functions of any humoral immune response post-immunization. When examining the ability of serum to recognize native Pvs25 by IFA, sera from all regimes resulted in surface staining, corresponding to surface localization of Pvs25 [18], [40]. Staining was not observed with sera collected from mice immunized with control regimes (regimes 5, 6, 7 and 8), or when IFAs were conducted on non-Pvs25-expressing (WT 2.34) ookinetes.

Although high titers are desirable, the functional effect of immunization on intensity and prevalence remains the key readout to measure TBV effectiveness. These experiments employed a proven transgenic model [18], [19], [40] to compare the ability of immunization to reduce oocyst development in the mosquito, giving *in vivo* data to examine the comparative potency of potential Pvs25-targeted TBVs. Every Pvs25-derived immunization regime examined induced a TB response, with varying efficacy. In this study, immunization with the Pvs25-ChAd63/MVA platform (regime 3) led to the lowest observed TB effect (30.0% reduction in intensity/35.6% reduction in prevalence). Substitution of MVA boost at 8 weeks with 50 μg of Pvs25-FhCMB plus Abisco-100 adjuvant not only increased the end-point anti-Pvs25 antibody titer (against both Pvs25-FhCMB and r-Pvs25), but also increased the efficacy of the regime, to a 74.5% reduction in intensity/68.3% reduction in infection prevalence. The TB responses generated following this regime are the highest induced within this comparative study, clearly demonstrating the attractiveness of combining adenoviral delivery with a protein antigen*.* In the absence of adenoviral delivery, a potent response is still induced by regime 1, prime/boost with Pvs25-FhCMB/Abisco-100, generating the second highest titers and TB responses (65.25% reduction in intensity/54.1% reduction in prevalence). When using Alhydrogel as adjuvant, a 56.58% reduction in intensity/56.82% reduction in prevalence was recorded. Significant differences in antibody titers or TB efficacy were not recorded when using Alhydrogel or Abisco-100 as an adjuvant with Pvs25-FhCMB. None of the regimes generated a non-specific TB effect following challenge with *P. berghei* 2.34, suggesting a specific response against Pvs25, and a lack of non-specific anti-*P. berghei* TB activity. The TB potential of regime 4 (Pvs25-ChAd63/Pvs25-FhCMB – the highest performing regime in the DFA), was further evaluated by DMFA, resulting in a mean 85.3%/25.5% reduction in intensity and prevalence respectively, confirming the ability of antibodies generated by this regime to block transmission in field relevant assays. These results are broadly comparable to results observed using serum generated from i.m. delivery of adenoviral-vectored Pvs25 in mice, described in Miyata et al. [39], where DMFA (carried out in identical conditions to those described here) resulted in reported reductions in oocyst intensity and prevalence of 84.1% and 41.7%, respectively. These promising results were achieved with an immunization of 50 μg of Pvs25-FhCMB per mouse. Obviously, further dose ranging studies examining proportional immunizations will need to be conducted before perusing product development in human subjects.

Immunization with the regimes outlined here can be compared to previously reported studies where the transgenic chimeric parasite Pvs25DR3 was used to determine *in vivo* potency of candidate TBVs. Pvs25 produced using an experimental baculovirus expression system was shown to induce a strong TB effect of 83.8% (intensity) and 88.4% (prevalence) in DFA following *i.m.* immunization (mean control intensity of 2.16 oocysts/midgut) [19]. These results are comparable to the 74.5% (intensity)/68.3% (prevalence) reduction seen using regime 4 (Pvs25-ChAd63/Pvs25-FhCMB boost), but are higher than the efficacy observed with other immunogens and adjuvants used within this study. Crucially, the Baculovirus-Dual-Expression system (BDES) described in Blagborough et al. [19] is not currently approved for use in human trials, whereas both adenoviral and *Nicotiana*-based expression systems have an extensive track record for the production of antigens to be used in human trials. Additionally, a similar approach has previously been taken to examine the efficacy of adenoviral anti-Pfs25 (*P. falciparum* homolog of Pvs25) vaccines [38]. TB efficacy was assessed using a homologous chimeric parasite (Pfs25DR3) generated identically to Pvs25DR3. Cohorts of mice were immunized with AdHu5/MVA Pfs25 in a heterologous prime boost regime, and subjected to DFA. A 67% reduction in intensity and a 28% reduction in prevalence were reported. This translated to a 96% reduction in intensity and 78% reduction in prevalence at sera dilutions of one in five in SMFA. The ability of the plant-derived antigens tested in this study to induce *in vivo* and *ex vivo* efficacy similar to previously examined P25-based immunogens lends weight to their continued development within the TBV pipeline.

This study gives a clear indication as to the value of *Nicotiana* and adenoviral-derived immunogens to induce a TB response. The efficient plant-based production of Pvs25, generation of high titers and induction of a TB response demonstrates the capability of these approaches to produce anti-malarial TBVs. Crucially, this study also demonstrates that combining this with other vaccine platforms (e.g. adenoviral delivery) can elicit maximal potency.

## Conflicts of interest statement

The authors are not aware of any conflicts of interest arising from this work.

## Figures and Tables

**Table 1a:** Evaluation of TB activity induced by active immunization in DFA: Mice were immunized with Pvs25-derived immunogens (or controls) as shown in Fig. 2. Each regime contained 10 immunized mice and was sub-divided into two groups, each containing five mice. Each group was then challenged with *P. berghei* Pvs25DR3 (5 mice) or WT *P. berghei* 2.34 (five mice), and used to assess transmission to mosquitoes via DFA. Each mouse was injected with 10^6^ parasites. Mosquito midguts were dissected 10–12 days post feed. Mean intensities and prevalence were calculated from all mice. (a) For both regimes 2 and 4 with WT *P. berghei* challenge, mice developed cerebral malaria and were humanely culled before DFA could be performed, resulting in only four mice per group. Overall transmission blockade (in terms of both infection intensity and prevalence) was calculated by comparison to mice in the relevant immunized control groups. (b) Significance was assessed using Mann–Whitney *U*-test (to examine the difference in mean oocyst intensity) and the Fisher's exact probability test (to examine the difference in infection prevalence) (*p* < 0.05). Following challenge with WT *P. berghei* 2.34, no significant changes in either intensity or prevalence were observed with any immunization regime. Significant inhibition was only observed following challenge with Pvs25DR3. a = *p* < 0.05, Mann–Whitney *U*-test; b = *p* < 0.05, Fisher's exact probability test. Table 1 Evaluation of transmission-blocking activity by active immunization and DFA (a). Transmission following immunization, parasite challenge and DFA.

	Challenge	Total mosquitoes	Mean infection intensity (±SEM)	Mean infection prevalence
**Regime 1:** Pvs25-FhCMB Abisco-100	*Pb* Pvs25DR3 challenge (*n* = 5)	*n* = 248	0.48 (0.18)	18.6%
	*Pb* 2.34 challenge (*n* = 5)	*n* = 171	40.8 (5.10)	96.8%
**Regime 2:** Pvs25-FhCMB Alhydrogel	*Pb* Pvs25DR3 challenge (*n* = 5)	*n* = 199	0.66 (0.29)	19.6%
	*Pb* 2.34 challenge (*n* = 4)	*n* = 135	40.7 (5.90)	95.3%
**Regime 3:** Pvs25-ChAd63 - MVA	*Pb* Pvs25DR3 challenge (*n* = 5)	*n* = 226	0.99 (0.39)	25.8%
	*Pb* 2.34 challenge (*n* = 4)	*n* = 148	45.1 (6.10)	93.6%
**Regime 4:** Pvs25-ChAd63 - Pvs25-FhCMB -Abisco-100	*Pb* Pvs25DR3 challenge (*n* = 5)	*n* = 250	0.39 (0.17)	13.2%
	*Pb* 2.34 challenge (*n* = 5)	*n* = 142	42.8 (5.73)	98.0%
**Regime 5:** LicKM-Abisco-100	*Pb* Pvs25DR3 challenge (*n* = 5)	*n* = 255	1.37 (0.40)	40.4%
	*Pb* 2.34 challenge (*n* = 5)	*n* = 129	43.7 (8.66)	90.8%
**Regime 6:** LicKM-Alhydrogel	*Pb* Pvs25DR3 challenge (*n* = 5)	*n* = 239	1.51 (0.47)	39.7%
	*Pb* 2.34 challenge (*n* = 5)	*n* = 175	40.2 (5.80)	88.6%
**Regime 7:** GFP-ChAd63 - MVA	*Pb* Pvs25DR3 challenge (*n* = 5)	*n* = 227	1.59 (0.54)	40.1%
	*Pb* 2.34 challenge (*n* = 5)	*n* = 167	38.0 (5.14)	94.0%
**Regime 8:** GFP-ChAd63–LicKM-Abisco-100	*Pb* Pvs25DR3 challenge (*n* = 5)	*n* = 214	1.54 (0.51)	41.7%
	*Pb* 2.34 challenge (*n* = 5)	*n* = 134	41.8 (6.1)	93.3%

**Table 1b:** Evaluation of transmission-blocking activity by immunization and DFA.

	Challenge	Mean change in infection intensity	Mean change in prevalence
Regime 1: Pvs25-FhCMB Abisco-100	*Pb* Pvs25DR3 challenge (*n* = 5)	65.3% ^a^	54.1% ^b^
	*Pb* 2.34 challenge (*n* = 5)	6.5%	-6.6%
Regime 2: Pvs25-FhCMB Alhydrogel	*Pb* Pvs25DR3 challenge (*n* = 5)	56.6% ^a^	51.6% ^b^
	*Pb* 2.34 challenge (*n* = 5)	-1.5%	-7.6%
Regime 3: Pvs25-ChAd63 - MVA	*Pb* Pvs25DR3 challenge (*n* = 5)	38.0%	35.6% ^b^
	*Pb* 2.34 challenge (*n* = 5)	-18.7%	0.42%
Regime 4: Pvs25-ChAd63 - Pvs25-FhCMB -Abisco-100	*Pb* Pvs25DR3 challenge (*n* = 5)	74.5% ^a^	68.3% ^b^
	*Pb* 2.34 challenge (*n* = 5)	-2.4%	-4.9%
